# Fenoldopam Increases Urine Output in Oliguric Critically Ill Surgical Patients

**DOI:** 10.7759/cureus.20445

**Published:** 2021-12-15

**Authors:** Joaquin A Cagliani, Laura Marinelli, Youngmin Cho, Santiago J Miyara, Andres Ruhemann, Andre Loyola, Ernesto Molmenti, Candace Smith, Gene Coppa, Rafael Barrera

**Affiliations:** 1 Anesthesiology, SUNY Downstate Medical Center, New York, USA; 2 General Surgery, Northwell Health, New York, USA; 3 Internal Medicine, Northeast Georgia Medical Center Gainsville, Georgia, USA; 4 Emergency Medicine, Northwell Health, New York, USA; 5 Internal Medicine, Medical Research Institute Alfredo Lanari, Buenos Aires, ARG; 6 Transplant Surgery, Northwell Health, New York, USA; 7 College of Pharmacy and Health Sciences, St. John’s University, New York, USA

**Keywords:** fenoldopam, acute kidney injury, urine output, diuretics, sicu, perioperative management, oliguria

## Abstract

Background

Fenoldopam is a short-acting dopamine A1 receptor agonist which mediates vasodilation of the renal arteries, thereby increasing urine output. The objective of this study was to compare the effects of fenoldopam and its synergistic effect on furosemide for improving the urine output in postoperative critically ill patients with acute kidney injury (AKI).

Methods

This is a retrospective study of postoperative critically ill patients with AKI. Patients who received furosemide (control group) were compared with those who received furosemide plus fenoldopam (treatment group) and evaluated at 12 and 24 hours post-treatment. Patients with oliguria and AKI were included in the study, while patients with chronic kidney disease (CKD) were excluded. Glomerular filtration rate, serum creatinine, blood pressure, calculated fluid accumulation, fluid intake, urine output, and total fluid output were used as variables to assess the medication effect.

Results

Of the 126 patients who met the inclusion and exclusion criteria, 87 patients received furosemide alone, and 39 patients received furosemide plus fenoldopam during their first 24 hours of admission to the surgical intensive care unit (SICU). Although not statistically significant, the addition of fenoldopam demonstrated an increase in mean urine output of 1525ml (IQR; 1530-2095) in the first 24 hours (P=0.06). There was also noted an increase in the urine output (p= 0.07) and a decrease in the total fluid accumulation when fenoldopam was co-administered with furosemide when compared to the patients who were only treated with furosemide (p=0.06). There was no significant change in creatinine clearance from baseline in either group.

Conclusion

Fenoldopam may increase urine output in postoperative critically ill patients with acute kidney injury when administered within the first 24 hours of presentation. Based on our results, fenoldopam appears to have a synergistic effect with furosemide in our study population.

## Introduction

Postoperative patients tend to receive a significant amount of fluid in order to maintain hemodynamic stability. However, fluid accumulation could cause drastic consequences such as pulmonary, cerebral, or intestinal edema due to low physiologic fluid control and comorbidities [1]. Patients with fluid accumulation and acute kidney injury (AKI) have a higher mortality, with previous studies showing a 60-day mortality rate of 36% compared to 16% when fluid accumulation is not an issue [2,3].

Since the unified use of Risk, Injury, Failure, Loss, and End-stage (RIFLE) criteria from the Acute Dialysis Quality Initiative of 2004, the overall incidence range of AKI has increased to 20% to 50%. However, in 2012 the Kidney Disease Improving Global Outcomes (KDIGO) released their clinical practice guidelines for acute kidney injury (AKI), which build off of the RIFLE criteria. 

Patients who have a high risk of AKI have comorbidities that further place them at risk for developing residual renal dysfunction that increases long-term mortality or progression to end-stage renal disease [3-6]. Many incidences of AKI in surgical patients should be preventable, but the mechanism of AKI is not fully understood, and many clinical trials for preventative strategies had discouraging results [7,8]. Therefore, causing patients to have a longer length of hospital stay and greater incidence of re-hospitalization. Thus, increasing the cost and strain to the healthcare system [3,7,9].

Surgical patients are at risk for acute kidney injury, many of whom develop residual renal dysfunction that increases fluid accumulation, long-term mortality, and the risks of progression to end-stage renal disease. Patients in the surgical intensive care unit have comorbidities that further place them at risk for developing AKI after surgery. Only 30% of those are preventable. Unfortunately, the preventative strategies such as discontinuing angiotensin-converting enzyme inhibitors (ACEI) and angiotensin receptor blockers (ARBs) preoperatively do not target the biological mechanism responsible for AKI nor increase kidney perfusion [10].

Fenoldopam, a dopamine-1 (D-1) receptor agonist and rapid vasodilator, increases renal perfusion and glomerular filtration rate without significantly altering peripheral blood pressure. Therefore, fenoldopam may potentially improve morbidity and mortality in surgical patients with acute kidney injury. In renal vasculature, activation of D-1 receptors causes vasodilation of glomerular arterioles. It has been postulated that D-1 receptors may also increase sodium excretion, subsequently increasing urinary output [1]. According to a meta-analysis of eighty-three publications, fenoldopam significantly reduced postoperative AKI in patients in the intensive care unit, including those with pre-existing kidney disease [7].

Thus, we wanted to focus only on those patients in the intensive care unit that, under the standard RIFLE criteria, were diagnosed with AKI. The aim of our study was to determine whether low-dose fenoldopam in synergism with furosemide could increase the urine output in patients with acute kidney injury in order to prevent fluid accumulation. 

## Materials and methods

This was an IRB-approved, retrospective chart review of adult patients with AKI admitted to the intensive care unit who received either furosemide plus fenoldopam (treatment group) or only received furosemide (control group). We identified 126 patients from January 2016 to October 2016 from a single tertiary care university hospital based on our selection criteria. We excluded those patients with pre-established chronic kidney disease (CKD) or in dialysis based on their clinical records and included those patients with oliguria (less than 500 milliliters of urine output in the last six-hour period) and AKI. RIFLE criteria was used to monitor the progression of AKI severity for the duration of hospitalization. The Cockcroft-Gault equation was used to determine the creatinine clearance at 24 hours. Serum creatinine at 24 hours and 48 hours post-treatment were also collected. Other variables such as blood pressure, central venous pressure, fluid intake at baseline, and urinary output were also measured at 12 and 24 hours. The fluid accumulation calculation was performed 24 hours post-admission to the intensive care unit. Both group variables were registered and were documented in patients with or without injury/failure according to age, sex, serum creatinine, fluid balance, fluid accumulation, urine output, and creatinine clearance.

Data are expressed as median (interquartile range) for non-normal distributed quantitative variables and as numbers and percentages for categorical variables. The normality of the distribution was determined using the Shapiro-Wilk test. The association between the group of patients who received furosemide and the ones who received furosemide plus fenoldopam was analyzed using the Mann-Whitney test given the non-normal distribution. A two-tailed p-value <0.05 was considered statistically significant. Statistical analysis was performed using the SAS system (SAS Institute, Cary, North Carolina) [11].

## Results

Among the 126 patients who met inclusion criteria, 87 patients received furosemide alone, and 39 patients received the combination of fenoldopam and furosemide in the first 24 hours. Both groups were classified in injury (I) (<0.5ml/kg/hr*12) or failure (F) (<0.3ml/kg/hr*24) according to RIFLE criteria within 24 hours in the intensive care unit.

The variables of groups were registered and documented in patients with RIFLE-I or -F or RIFLE risk (R) according to age, sex, and weight (Table 1). No significant differences were found between them. Among 87 patients, 38 were males, and 53 were females with furosemide alone, the age range between 30 to 95 years old and it was not significant between groups. In terms of weight, the median and IQR were 76.8 kg (59.1kg-91.8kg) for the RIFLE-I or -F, while 74.4kg (65.0kg-82.6kg) in the RIFLE-R group (p=0.48). Alternatively, in the furosemide + fenoldopam group, there were 19 male and 20 female patients, the median age was 80 (73-86) years old in RIFLE-I and-F and 71 (67-77) years old in the RIFLE-R group, the median weight was 80.5kg (73.0kg-90.3kg) for RIFLE-R group, and 69.9 kg (60.8kg-88.5kg) for RIFLE-I or -F group (p=0.37).

**Table 1:** Demography of the study subjects RIFLE - Risk, Injury, Failure, Loss, and End-stage criteria

	Furosemide group		Furosemide plus fenoldopam group	
	RIFLE- I or -F (n=48)	RIFLE-R (n=39)	RIFLE- I or -F (n=20)	RIFLE-R (n=19)
Median (IQR 25th-75th) age (years)	77 (63-86)	75 (62-83)	80 (73-86)	71 (67-77)
Gender (females %)	31 (67%)	22 (56%)	9 (45%)	11 (57%)
Median (IQR 25th-75th) weight (kg)	76.8 (59.1-91.8)	74.4 (65.0-82.6)	69.9 (60.8-88.5)	80.5 (73.0-90.3)

Patients who were administered furosemide alone received 2598ml (2032ml-4981ml) of IV fluids within 24 hours in the RIFLE-R group while 4197ml (2881ml-7313ml) for patients with RIFLE-I or -F. Similarly, patients who were administered furosemide plus fenoldopam received 3320ml (2685ml-4479ml) of IV fluids in the RIFLE-R group (p=0.56) and 3965ml (2728ml-6263ml) in RIFLE-I or -F group (p=0.47 ) (Table 2).

**Table 2:** Median volume that patients received within 24 hours Both groups are classified in injury (<0.5ml/kg/hr*12) or failure (<0.3ml/kg/hr*24) according to RIFLE criteria within 24 hours in the intensive care unit. * p-value (0.07); ** p-value (0.06); Abbreviation: (FO%) - Fluid accumulation; IQR - Interquartile range; RIFLE - Risk, Injury, Failure, Loss, and End-stage

	Furosemide group		Furosemide plus fenoldopam group	
	RIFLE-I or F (n=48)	RIFLE-R (n=39)	RIFLE-I or -F (n=20)	RIFLE-R (n=19)
Creatinine serum 24 hrs. (mg/dL) Median (IQR 25th-75th)	1.22 (0.82-1.79)	1.37 (0.91-1.77)	1.33 (0.86-2.51)	1.06 (0.6-1.84)
Creatinine serum 48 hrs.(mg/dL) Median (IQR 25th-75th)	1.28 (0.86-1.98)	1.24 (0.79-1.84)	1.47 (0.83-2.94)	1.20 (0.59-1.81)
IV Fluids within 24 hrs (ml) Median (IQR 25th-75th)	4197 (2881-6313)	2598 (2032-4981)	3965 (2728-6263)	3320 (2685-4478)
Urine Output first 12 hrs (ml) Median (IQR 25th-75th)	205 (137-300)	782 (500-1725)	287 (170-483)	950 (535-1550)
Urine Output second 24 hrs (ml) Median (IQR 25th-75th)	320 (20-450)	865 (675-1205)	421 (163-545)	1025 (640-1325)
Creatinine clearance 24 hrs. (mg/dL) Median (IQR 25th-75th)	48.46 (30.4-82.1)	49 (29.6-98.7)	40.65 (20.6-73.30	66.50 (35.3-109.3)
Fluid balance 24 hrs (ml) Median (IQR 25th-75th)	3700 (146-5656)	1325 (675-2998)	3192 (1618-6565)	882 (372-3014)
Fluid Accumulate, FO% (ml) Median (IQR 25th-75th)	5179 (2121-7233)	1588 (774-3765)	4230 (2132-7843)*	1074 (612-3996)**

Although not statistically significant, an increase in the urine output was noted in the first 12 hours as well as during the second 24 hours in the patients who received furosemide plus fenoldopam in patients with RIFLE-I or -F (287ml [170ml-483ml] vs 205ml [137ml-300ml]; p=0.26), (421ml [163ml-545ml] vs 320ml [120ml-450ml]; p= 0.22) and also RIFLE R (950ml [535ml-1550ml] vs 782ml [500ml-1725ml]; p=0.18) (1025ml [640ml-1325ml] vs 865ml [675ml-1205ml]; p=0.24) groups when compared to the patients who only received furosemide respectively. Given the changes on urine output, a decrease in fluid accumulation was therefore found in the RIFLE-R between the patients who received furosemide + fenoldopam versus only furosemide (1588 ml [774ml-3765ml] vs 1074 ml [612ml-3996ml]; p=0.07). However, the effect was more pronounced in the patients with a RIFLE-I or F who were administered furosemide + fenoldopam versus only those patients who received only furosemide (5179 ml [2121ml-7233ml] vs 4230ml [2132ml- 7843ml]; p=0.06). The creatinine clearance did not show any significance between groups (p=0.53).

## Discussion

Fenoldopam is a short-acting dopamine A1 receptor agonist which presents widely in the kidney and dilates the renal arteries. This effect is greater in the medulla than the cortex, thereby increasing renal blood flow, creatinine clearance, urinary flow, and excretion of sodium as a result of blocking the sodium-potassium adenosine triphosphates-dependent in the proximal convoluted tubules and the thick part of the ascending loop of Henle increase the diuresis. Moreover, fenoldopam has a theoretical advantage against ischemia by inhibiting the tubular Na+ K+ adenosine triphosphate phase in the medulla and also decreasing the oxygen demand in the tissues [12,13]. Moreover, keeping a high flow in the tubes potentially decreases the risk of obstruction and limits the back-leakage. Other functions of fenoldopam include peripheral vascular vasodilation [2] and its anti-inflammatory properties in experimental models [5,7]. Low-dose fenoldopam (≤1 μg/kg/min) has been shown to improve renal function in AKI patients in the US and in Europe without significant systemic effects. However, when used above 1 μg/kg/min, fenoldopam is an effective, titratable antihypertensive medication [14]. In cardiac surgery, fenoldopam has shown some promising results as a renal protective agent in multiple case reports and randomized trials [3,13,15].

Patients in the intensive care unit often present with impaired organ recovery, disrupted wound healing, suboptimal rehabilitation secondary to positive fluid loss [15]. Thus, preventing fluid overload has a significant impact in decreasing mortality. In 2010 the European Society of Intensive Care Medicine Working Group for Nephrology published recommendations suggesting the prophylactic use of fenoldopam in patients at risk for AKI [16]. Fenoldopam also increases the urine output in the intensive care unit patients with AKI. A meta-analysis by Landoni et al. showed the beneficial effect of the prophylactic use of fenoldopam in cardiovascular patients at risk for AKI by reducing the AKI and renal replacement therapy events and the in-hospital mortality [8,12,15,17]. This statement was further supported in 2010 by the European Society of Intensive Care Medicine Working Group for Nephrology. Due to the physiologic benefits of fenoldopam as a renoprotective medication, as previously mentioned, we calculated the predictive clearance of creatinine according to the Cockcroft-Gault equation within 24 and 48 hours in patients with furosemide alone and furosemide plus fenoldopam. The P values were 0.88 and 0.70, respectively. Despite the relative difference in the urine output, we did not find any statistical difference when adding fenoldopam (Table 2).

Patients with RIFLE criteria I-F being treated with furosemide alone had more fluid accumulation than those patients who were treated with furosemide plus fenoldopam. We believe that despite the lack of statistical significance between urine outputs of both groups, adding fenoldopam can decrease the risk of positive fluid accumulation. Indeed, the median fluid accumulation decreased by 508ml (13%) in patients who were treated with furosemide plus fenoldopam compared to those who were treated only with furosemide alone. We suggest that the effect of fenoldopam on oliguric intensive care unit patients should be further investigated with a multicenter, randomized, controlled trial, as this study suggests promising trends. In addition, identification of subsets of patients who respond well to fenoldopam would help to direct its use as a therapy for oliguria and renal failure.

Studies in neonatal patients after cardiac surgery revealed increment of the urinary output within 24 hours after receiving fenoldopam without any response of conventional diuretics or escalation of inotropic support [18,19]. Our study presents several limitations in regard to each of the three analyzed variables that should be outlined. First, this is a retrospective and single-center nature of the study. In addition, recent studies have shown that kinetic glomerular filtration rate estimation may have more power and versatility than the Cockcroft-Gault formula for evaluating kidney function in patients with AKI whose plasma creatinine fluctuates rapidly as compared with CKD [20-21]. Also, newer classifications such as the Kidney Disease Improving Global Outcomes (KDIGO) have been shown to be more was predictive for in-hospital mortality compared to the RIFLE classification. Therefore, the results of this study could potentially have a stronger statistical significance [22]. Finally, the study has a lack of power to run regression analysis that could help exclude confounding factors.

## Conclusions

Fenoldopam helps increase the urine output in patients with AKI in the intensive care unit within 24 hours, and it has an added effect to diuretics like furosemide at 24 hours in patients with AKI. The effects on decreasing the peripheral vascular resistance, along with increasing renal blood flow, diuresis and natriuresis, can be beneficial in critically ill patients given its anti-hypertensive and renal-sparing properties.
